# An acellular biologic scaffold treatment for volumetric muscle loss: results of a 13-patient cohort study

**DOI:** 10.1038/npjregenmed.2016.8

**Published:** 2016-07-21

**Authors:** Jenna Dziki, Stephen Badylak, Mohammad Yabroudi, Brian Sicari, Fabrisia Ambrosio, Kristen Stearns, Neill Turner, Aaron Wyse, Michael L Boninger, Elke H P Brown, J Peter Rubin

**Affiliations:** 1 McGowan Institute for Regenerative Medicine, University of Pittsburgh, Pittsburgh, PA, USA; 2 Department of Bioengineering, University of Pittsburgh, Pittsburgh, PA, USA; 3 Department of Surgery, University of Pittsburgh, Pittsburgh, PA, USA; 4 Department of Physical Therapy, University of Pittsburgh, Pittsburgh, PA, USA; 5 Department of Rehabilitation Sciences, Jordan University of Science and Technology, Al Ramtha, Irbid, Jordan; 6 Department of Physical Medicine & Rehabilitation, University of Pittsburgh, Pittsburgh, PA, USA; 7 Department of Radiology, University of Pittsburgh, Pittsburgh, PA, USA; 8 Department of Plastic Surgery, University of Pittsburgh, Pittsburgh, PA, USA

## Abstract

Volumetric muscle loss (VML) is a severe and debilitating clinical problem. Current standard of care includes physical therapy or orthotics, which do not correct underlying strength deficits, and surgical tendon transfers or muscle transfers, which involve donor site morbidity and fall short of restoring function. The results of a 13-patient cohort study are described herein and involve a regenerative medicine approach for VML treatment. Acellular bioscaffolds composed of mammalian extracellular matrix (ECM) were implanted and combined with aggressive and early physical therapy following treatment. Immunolabeling of ultrasound-guided biopsies, and magnetic resonance imaging and computed tomography imaging were performed to analyse the presence of stem/progenitor cells and formation of new skeletal muscle. Force production, range-of-motion and functional task performance were analysed by physical therapists. Electrodiagnostic evaluation was used to analyse presence of innervated skeletal muscle. This study is registered with ClinicalTrials.gov, numbers NCT01292876. *In vivo* remodelling of ECM bioscaffolds was associated with mobilisation of perivascular stem cells; formation of new, vascularised, innervated islands of skeletal muscle within the implantation site; increased force production; and improved functional task performance when compared with pre-operative performance. Compared with pre-operative performance, by 6 months after ECM implantation, patients showed an average improvement of 37.3% (*P*<0.05) in strength and 27.1% improvement in range-of-motion tasks (*P*<0.05). Implantation of acellular bioscaffolds derived from ECM can improve strength and function, and promotes site-appropriate remodelling of VML defects. These findings provide early evidence of bioscaffolding as a viable treatment of VML.

## Introduction

Volumetric muscle loss (VML) as a result of tumour ablation, trauma or disease remains a challenging clinical problem for which therapeutic options are limited. Current noninvasive treatment for VML includes maximising strength of remaining muscle and bracing. Unfortunately, this approach cannot make up for the lost strength associated with VML. Muscle transposition or tendon transfer can replace muscle function, but have less than satisfactory success rates.^1–4^ Such procedures typically involve significant donor site morbidity and fail to provide efficient reconstruction or functional re-innervation of the lost muscle tissue. These approaches often result in persistent strength and functional deficits, which contribute to disability, weakness and compromised quality of life for patients with VML.

Skeletal muscle retains a limited capacity to regenerate following a severe acute injury. The regenerative process is dependent on resident progenitor cell populations, including satellite cells and myoblasts, which have the potential to proliferate and differentiate into functional myofibers. Cell-based regenerative medicine strategies have attempted to augment this regenerative process through the delivery of exogenous (typically autologous) stem/progenitor cells to the VML defect site. Utilisation of enriched muscle-derived stem cells, capable of long-term proliferation and myogenic potential, has been somewhat successful and has been shown to increase the regenerative index when injected into sites of skeletal muscle injury.^5–7^ Such approaches are limited, however, by issues of low cell viability,^8^ poor cell migration and engraftment, and the need for immunosuppressive therapy, among others.^9,10^ In fact, immunosuppressive therapy can further contribute to myoblast apoptosis.^11^ Even if an ideal cell source and an effective delivery method are utilised, transplanted cells are often associated with less than optimal proliferative and differentiation potential within the host injury site.^12^ Cell-centric strategies are also associated with high cost due to the need for *ex vivo* cell expansion and manipulation. While some cell-based approaches have shown promise in preclinical studies, regulatory challenges and a lack of notable efficacy have prevented their widespread adoption of treatment for VML.^13^

We recently described an acellular bioscaffold approach for treatment of VML in five patients that showed encouraging results.^14^ This approach involved the use of extracellular matrix (ECM) derived from decellularized porcine urinary bladder to promote scaffold-associated skeletal muscle tissue formation and partial restoration of function. ECM bioscaffold implantation was also associated with the recruitment of endogenous perivascular stem cells (PVSCs). While ECM bioscaffolds have been used in reconstructive surgery, they are typically employed only as a barrier or reinforcing layer of soft tissue. In our prior report,^14^ we provided evidence for functional remodelling of the ECM scaffold with formation of new muscle tissue. An aggressive early post-operative rehabilitation protocol was a component of this strategy to place dynamic strain on the ECM and contribute to site-appropriate differentiation of the recruited stem/progenitor cells. The mechanism(s) of action responsible for ECM bioscaffold-mediated VML repair are partially understood and include host cell-mediated scaffold degradation and recruitment of endogenous progenitor cells.^14–17^ The recruitment of neurogenic cells and modulation of the innate immune response are also considered as common features associated with ECM-mediated constructive remodelling in preclinical studies.^18–20^ Overall, ECM bioscaffolds have been shown to stimulate endogenous repair.^21^

The present manuscript describes the results from the first 13 patients treated using the acellular bioscaffold approach, including results from the first 5 patients previously reported.^14^ The results reported herein advance the previously reported findings in several respects: first, it expands the number of patients and the anatomic sites of VML subjected to treatment; second, it includes the use of three different source tissues of ECM bioscaffolds; third, it includes the investigation of neurogenic cells as a component of the functional remodelling process; and finally, it includes electrodiagnostic evaluation of the remodelled muscle tissue.

## Results

### Biologic scaffold implantation for the treatment of VML is associated with increased skeletal muscle force production

Thirteen subjects with VML were enroled in this cohort study and the average tissue deficit for all patients was 66.2%, when compared with the contralateral limb (Table 1). All subjects met established inclusion criteria (Supplementary Table S1) and had received standard of care options, including surgical intervention and/or physical therapy. Strength testing showed that 7 of 13 patients had improvement from their pre-surgical maximum strength as early as 6–8 weeks after surgery, by an average of 15.2%±12.6 with a maximum of 127.9% and a minimum of −33.3% (Table 2). By 10–12 weeks, patients showed an average change of 21.1%±12.2 with a maximum of 149.2% and a minimum of −33.0%. At 24–28 weeks, patients showed an average force production change of 37.3%±12.4 with a significant improvement when compared with pre-operative measurements (*P*<0.05), with a maximum of 136.1% and a minimum of −17.88%

### Biologic scaffolds for VML treatment are associated with improved range-of-motion and functional outcomes

Tasks to assess range-of-motion were performed and data is reported for all patients who showed range-of-motion deficits pre-operatively. At 6–8 weeks post surgery, all tested subjects showed improvement in at least one range-of-motion task with an average change of 16.7%±4.9. At 10–12 weeks, average range-of-motion change compared with pre-operative measures was significantly increased (*P*<0.05) at 24.0%±6.8. By 24–28 weeks after surgery, this improvement increased to 27.1%+10.5 (*P*<0.05) (Supplementary Table S3).

**Figure 1:**
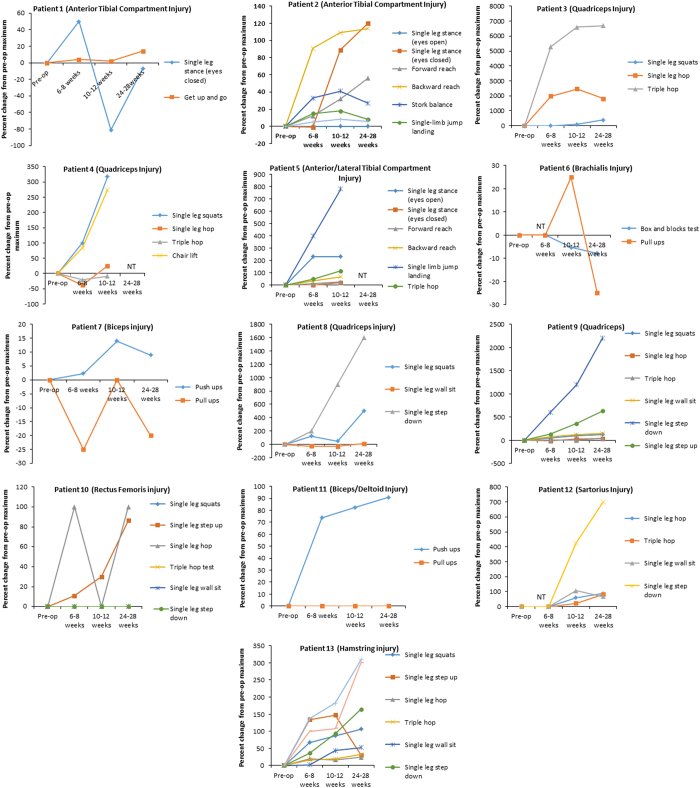
Functional task performance. Functional measures as assessed by task/exercise completion from each patient. Data represent per cent change from pre-surgical maximum. NT, not tested.

At 6–8 weeks post surgery, 10 out of 13 patients showed a ⩾20% improvement in performance of at least one functional task when compared with pre-surgical performance (range, 20–1980%). By 10–12 weeks, 12 of 13 patients showed a ⩾20% improvement (range, 20–2460%), and by 24–28 weeks 9 of 13 patients showed a ⩾20% improvement (range 25–1,820%). Patient 3 showed particularly notable improvement in the single-leg hop test, improving by 1,980%, 2,460% and 1,820% at 6–8, 10–12 and 24–28 weeks after surgery, respectively (Supplementary Table S2). Patient 5 showed a dramatic increase in single-leg jump landing distance, improving by 400%, 783.3% and 1,050% at 6–8, 10–12 and 24–28 weeks after surgery, respectively (Supplementary Table S2). Likewise, patient 8 showed improvements in the single-leg step down task of 200, 900 and 1,600% (Figure 1). Twelve of 13 patients showed improvement in at least 1 functional task by 24–28 weeks after surgery.

### ECM bioscaffold implantation is associated with PVSC mobilisation, electromyographic evidence of innervation, the presence of neurogenic cells at the remodelling site and new muscle formation

**Figure 2:**
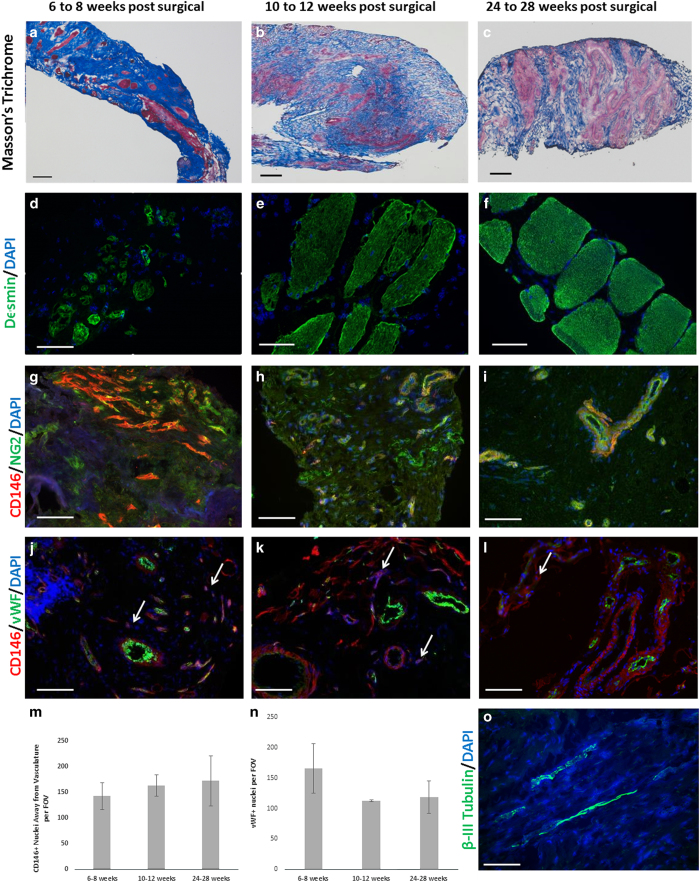
Site-appropriate tissue remodelling by ECM bioscaffolds. (**a**–**c**) Massons trichrome staining of human muscle biopsies shows islands of skeletal muscle present at 6–8 weeks, 10–12 weeks and 24–28 weeks post surgery, respectively. (**d**–**f**) Human muscle biopsies are characterised by desmin expression at all time points, indicating new muscle formation within the site of implantation. (**g**–**i**) ECM bioscaffold implantation is associated with the presence of CD146+NG2+ perivascular stem cells. (**j**–**l**) PVSCs were shown to migrate away from their normal vessel-associated anatomic location at all time points. Arrows indicate CD146+ PVSCs migrating away from vessels. (**m**, **n**) Migrating PVSCs and vascularity was quantified using CellProfiler image analysis software. (**o**) At 24–28 weeks post surgery, ECM bioscaffold implantation was associated with the presence of β-III tubulin+ cells, implicating innervated skeletal muscle. (Scale bars=50 μm).

**Figure 3:**
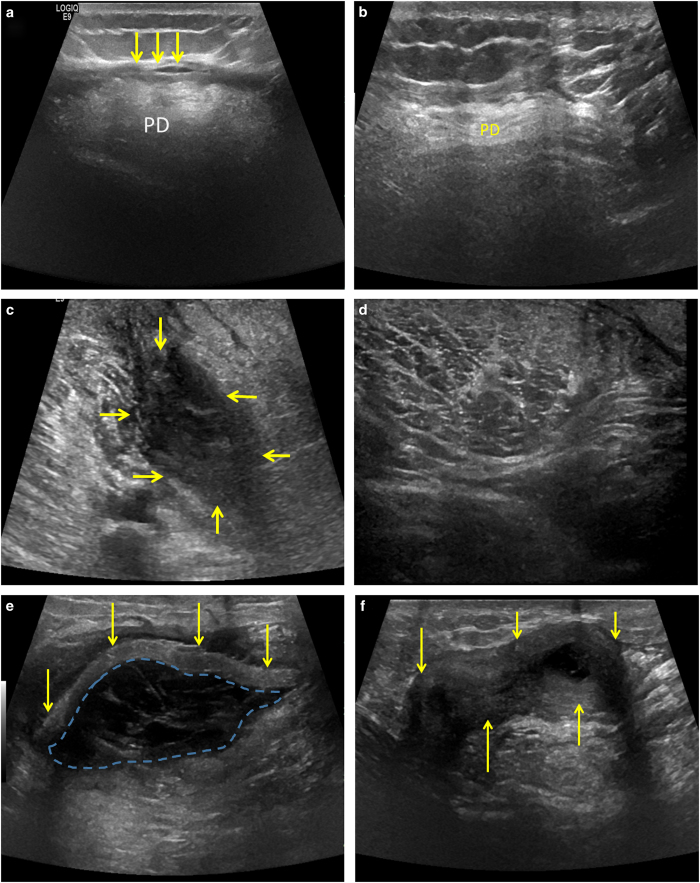
Ultrasound imaging shows that ECM bioscaffolds degrade on implantation. (**a**) Grayscale ultrasound image 1 month after surgery in the posterior shoulder demonstrates a thin, sheet-like hyperechoic structure representing SIS-ECM (yellow arrows) overlying the posterior deltoid muscle. The posterior deltoid muscle is increased in echogenicity due to underlying fatty infiltration. (**b**) Ultrasound imaging 7 months after surgery shows that surgically-placed SIS-ECM is not longer identifiable superficial to the posterior deltoid. (**c**). Ultrasound image 1 month after surgery in the medial mid thigh demonstrates an ill-defined hypoechoic structure representing SIS-ECM (yellow arrows) adjacent to the sartorius muscle. (**d**) Ultrasound image 7 months after surgery shows that surgically-placed SIS-ECM is no longer identifiable and the sartorius muscle appears to have enlarged. (**e**) Ultrasound imaging 1 month after surgery in the posterior mid thigh demonstrates a sheet-like echogenic structure representing dermal ECM (yellow arrows) with surrounding complex anechoic material (dashed-blue line) likely representing post-operative fluid collection. (**f**) Ultrasound imaging 7 months after surgery shows dermal ECM (yellow arrows) has decreased in echogenicity and now has a tubular or ‘rolled-up’ appearance as opposed to a sheet-like appearance. The previously identified post-operative fluid collection has essentially resolved.

Tissue biopsies of the remodelling implantation site were obtained at 6–8 weeks, 10–12 weeks and 24–28 weeks post surgery, and showed a robust mononuclear cellular infiltration into the bioscaffold site along with evidence of muscle formation as early as 6–8 weeks post surgery (Figure 2a), which was increased at each subsequent biopsy time point. Immunolabeling studies showed CD146+NG2+ PVSCs localised around vWF+ vessels at all time points (Figure 2g–i). PVSCs were also found removed from their normal anatomic location, suggesting their potential contribution to skeletal muscle formation (Figure 3j–l). Desmin+ cells with central nuclei were present as early as 6–8 weeks post surgery (Figure 2d–f) with striated desmin+ muscle fibres present in all biopsy samples at both 10–12 and 24–28 weeks after surgery (Figure 2e,f). These desmin+ muscle fibres were present at locations both near the interface with native uninjured muscle and within the centre of the scaffold site with no evidence for continuity with adjacent native healthy muscle. Biopsies also showed an increase in the presence of β-III Tubulin+ nerve bundles by 6 months after implantation throughout the scaffold implant site (Figure 2o). CellProfiler (Broad Institute, Cambridge, MA, USA) quantification showed no significant differences in the number of migrating PVSCs or vessels between time points (Figure 2m,n).

### ECM scaffolds degrade following implantation

Representative ultrasound imaging at 1 month after surgery showed a sheet-like hyperechoic structure consistent with the ECM scaffold overlying and adjacent to the native uninjured muscle (Figure 3a). By 7 months BioDesign (SIS-ECM) and Matristem (UBM-ECM) ECM scaffold materials were no longer identifiable (Figure 3b), whereas the Xenatrix (dermis-ECM) ECM scaffold was still identifiable (Figure 3f). In addition, increased muscle tissue, identified by an imaging signal consistent with muscle, was present at the site of ECM scaffold placement (Figure 3d).

### ECM treatment increases bulk muscle content

**Figure 4:**
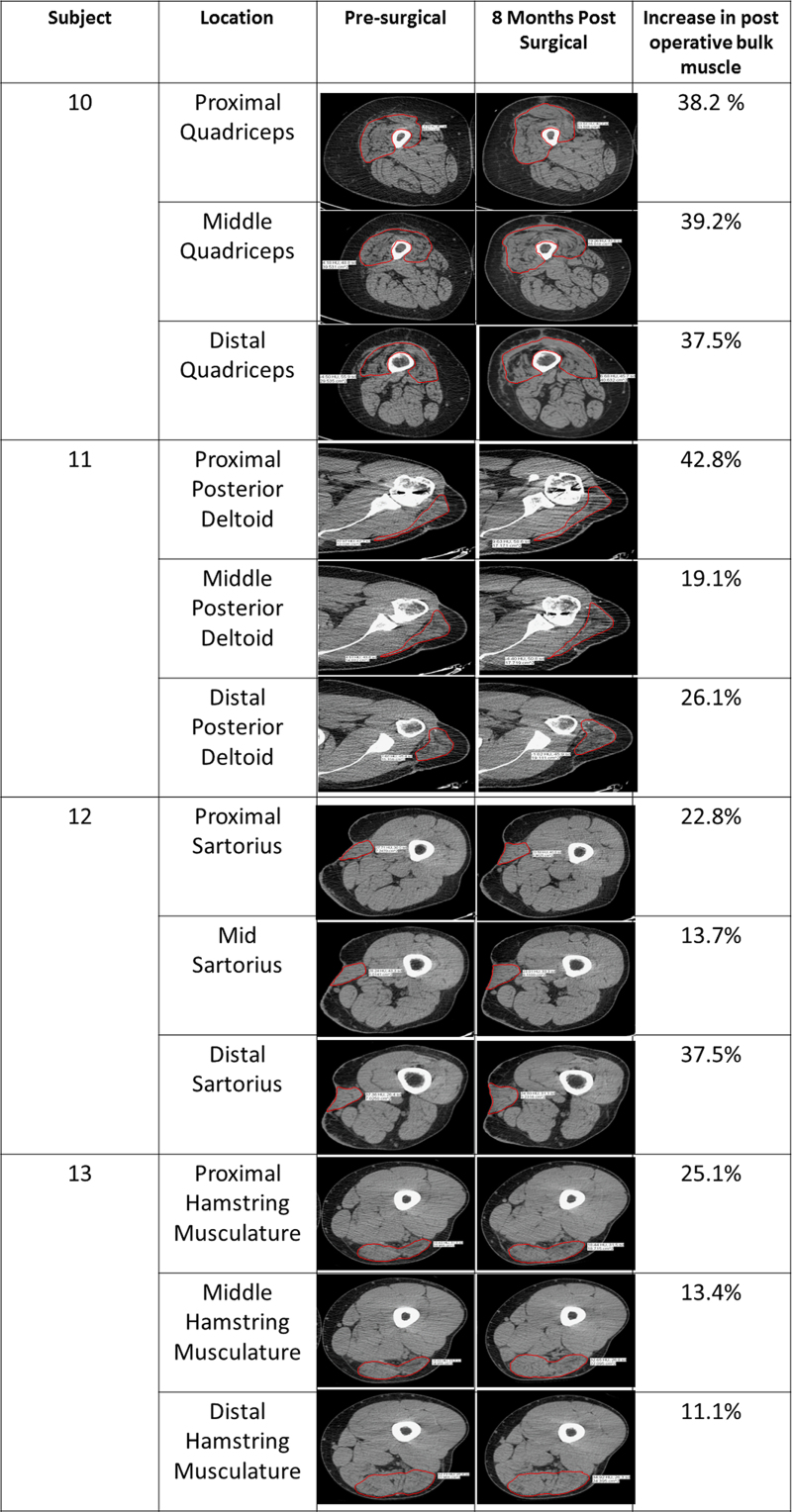
Representative CT imaging shows ECM bioscaffold implantation increases post-operative bulk muscle content. Overall area of the treated muscle was measured at three representative sites (proximal, middle and distal) both prior to surgery and 7 months after surgery in multiple anatomic locations.

Before surgery, the average per cent of muscle loss ranged from 25–90% of contralateral limb tissue (Table 1). By 8 months, computed tomography (CT) or magnetic resonance imaging (MRI) imaging showed an increase in dense tissue consistent with that of skeletal muscle within the implantation site. Post-operative muscle bulk was calculated by selecting a region-of-interest in CT or MRI images. Bulk muscle increased in all patients post-operatively, with an average increase of 27.2% (Figure 4). Interestingly, Patient 13 showed complete atrophy and absence of hamstrings due to rupture pre-operatively. Following ECM treatment, the implant site was replaced with tissue characterised by an imaging signal consistent with muscle at measurements of 5.45 cm^2^, 6.90 cm^2^ and 7.39 cm^2^ at the proximal, middle and distal aspect of the defect in the posterior compartment, respectively (Supplementary Figure S1).

### ECM bioscaffold implantation improves electrophysiological function

Electrodiagnostic studies were conducted on 8 of the 13 patients. At baseline, seven of the eight tested subjects presented with patterns of mononeuropathies, with three subjects with anterior compartment injuries in the lower leg presenting with deep peroneal mononeuropathy and three of the four subjects with quadriceps injury presenting with femoral mononeuropathy and one individual presented no abnormal finding. The abnormalities were limited to the injury site and did not extend distally along the nerve. Two tested subjects showed severe atrophy with undetectable compound motor action potentials (CMAPs). Post-operatively, four subjects increased CMAP amplitude: one in the tibialis anterior, two in the vastus medialis and one in the biceps brachii (Table 3). The remaining subjects showed no appreciable change in nerve conduction. Electromyography (EMG) analysis showed disappearance in abnormal spontaneous activity and improved recruitment patterning following ECM bioscaffold implantation (Table 4).

## Discussion

The present study provides a comprehensive analysis of the structural remodelling, strength and functional outcomes after VML defects were treated with ECM bioscaffolds in 13 human subjects. The study corroborates and extends the findings of previous work by not only increasing the number of patients, but also utilising three different forms of ECM bioscaffolds, identifying neurogenic cell types in the remodelling site, and documenting electrophysiologic evidence of innervation and its association with functional remodelling outcomes.

Acellular bioscaffolds for VML treatment represent an ‘off-the-shelf’ approach to muscle repair. As opposed to cell-based strategies, a bioscaffold approach obviates the requirements of cell isolation, manipulation, expansion, storage and delivery strategies. In the present study, ECM bioscaffolds derived from three different xenogeneic (porcine) source tissues—small intestinal submucosa (SIS), urinary bladder matrix (UBM) and dermal ECM—were utilised. Although our study was not powered to detect variation based on ECM, no differences in outcomes were seen based on bioscaffold used. These findings suggest the presence of similar signalling mechanisms within the ECM derived from three different tissues. A comparative analysis of the differences between ECM source tissues and their effects on skeletal muscle reconstruction could only be reliably performed in a very large sample due to the variety of anatomic sites at which they need to be placed. Each of the bioscaffolds used differ in their preparation methods including method of decellularization and terminal sterilisation. Such differences will logically confer differences in their mechanical and biochemical properties. Further investigation could provide insight into the preparation parameters that are associated with positive tissue remodelling outcomes and could implicate the specific bioscaffold constituents and/or properties that contribute to ECM-mediated skeletal muscle remodelling.

While the exact mechanism(s) by which ECM bioscaffolds promote constructive tissue remodelling are only partially understood, previous work has shown that their degradation on implantation generates low molecular weight matricryptic oligopeptides with the ability to recruit and influence endogenous progenitor cells.^22,23^ It has been shown that PVSCs play a role in ECM-mediated skeletal muscle repair.^14^ The present study shows CD146+NG2+ PVSCs are not only localised around their typical microvascular niche, but, following ECM implantation, mobilise away from this traditional anatomic site. All muscle biopsies showed this phenomenon, as well as evidence for neovascularization and the presence of site-appropriate desmin+ striated muscle as early as 6 weeks after bioscaffold implantation. The ability of ECM bioscaffolds to influence the local skeletal muscle injury microenvironment may allow for synergy and cross-talk between PVSCs, myoblasts, neuronal progenitors and other responding cell types, which contribute to skeletal muscle formation at the implant site. The presence of PVSCs and myoblasts within this site strongly suggests their participation in the remodelling process. Whether or not the behaviour of PVSCs and myoblasts is mediated directly by signalling from the ECM or via paracrine mechanisms is unknown; however, it is plausible and logical that other stem and progenitor cell populations also play a role in this constructive and functional remodelling process.

Desmin+ skeletal muscle fibres were found not only at the interface of the bioscaffold with native, adjacent, uninjured muscle, but also within the centre of the scaffold implantation site. The spatial distribution of skeletal muscle fibres clearly separated from the interface with adjacent uninjured native muscle suggests *de novo* skeletal muscle generation rather than simple integration of native muscle with the scaffold-filled defect site. *In vitro* studies have shown the ability of ECM signalling molecules to promote mitogenesis and myogenesis of skeletal muscle progenitor cells.^23^ The presence of β-III tubulin+ cells in association with these new islands of skeletal muscle, combined with positive EMG recordings, further supports the conclusion that functional islands of new skeletal muscle have been formed.

CT or MRI imaging corroborated the histologic findings showing an increase in post-operative soft tissue formation consistent with bulk skeletal muscle tissue in all 13 patients (Figure 4, Supplementary Figure 3). Whether or not this increase was due to an increase in the size or the number of muscle fibres requires further investigation. However, the needle EMG findings of decreased ASA and improved recruitment would seem to indicate new muscle fibre formation and gross changes in muscle appearance were evident (Supplementary Figure 2).

The histomorphologic and imaging studies were accompanied by clear and clinically relevant functional improvement. Two of the 13 patients showed an unappreciable change in force production compared with pre-operative outcomes, but 11 of 13 patients increased their pre-operative force production measured via dynamometer by 20–140% at 6 months after surgery. Twelve out of 13 patients showed improvement in functional task performance. It is important to note that all patients had previously undergone standard of care treatments, and custom designed, aggressive physical therapy regimens prior to ECM implantation and showed a plateau in force production or functional task performance. The improvements in performance following ECM bioscaffold implantation are thus likely due to ECM intervention. The importance of a rigorous physical therapy program following ECM implantation and its association with successful outcomes should not be underestimated. The application of a physiologic mechanical load (i.e., concomitant physical rehabilitation) during the entirety of the remodelling period following ECM implantation has been shown to promote favourable outcomes.^24–27^ It has been suggested that ECM bioscaffolds contribute to force improvement by simple force transduction based on results of a rodent model in which post-operative physical therapy could not be controlled.^28^ While the scar release of the procedure and the mechanical transduction effect of the ECM layer may both be contributing factors to the improved function, the histologic imaging and electrophysiologic evidence of vascularised, innervated skeletal muscle within the defect site in the present cohort of human patients suggests a positive and contributing role for new skeletal muscle in the functional outcomes. Taken together, the data from this study show that ECM implants for soft tissue reconstruction, while long regarded as a passive reinforcing layer, can undergo important functional remodelling during the healing process.

Electrodiagnostic studies conducted on 8 of the 13 patients showed concomitant nerve and muscle remodelling following ECM treatment. Specifically, seven subjects presented with a pre-operative electrodiagnosis of incomplete mononeuropathy in the area of the VML defect. After treatment with ECM, five of the eight patients showed improvements in nerve conduction or needle electromyography parameters including CMAP. These results indicate electrically active, functionally innervated muscle. Electrical activity present within the ECM implant site is consistent with a concomitant strength improvement. Histologic outcomes further corroborate these results showing presence of β-III tubulin+ cells within the remodelling site by 6 months after surgery.

The present study has several limitations. It was not possible to include an untreated control to determine the effects of scar tissue debridement and tenolysis alone. However, 12 of 13 patients had been subjected to extensive standard of care therapy (i.e., average of 10 previous surgeries across all patients) and failed to improve. Placebo effects (i.e., patients having more confidence after treatment, which could translate to improved functional outcomes) were uncontrolled. Although histologic outcomes show the presence of PVSCs and desmin+ muscle fibres within the ECM implantation site, and these findings were associated with improved functional and strength outcomes, the present study does not provide conclusive evidence that there is a causal relationship between the presence of these cells and the downstream functional improvements. The diverse nature of anatomic implant sites and physical therapy activities performed by the subjects made determination of an ‘average’ functional improvement following bioscaffold implantation impossible. No two patients had the same injury or comorbidities, and thus each had a personalised physical therapy regimen composed of specific exercises depending on the site of injury and other comorbidities.

The results of this 13-patient cohort study show that an acellular biologic scaffold approach can facilitate constructive and functional tissue remodelling following VML. The mechanisms by which such materials mediate their remodelling effects appear to include recruitment of myogenic progenitor cells, improved innervation and functional skeletal muscle formation. The findings reported herein support the use of ECM bioscaffolds as a viable treatment option for VML treatment.

## Materials and methods

### Overview of study design

A cohort study examining functional and histomorphologic outcomes following VML repair with acellular biologic scaffolds was conducted with informed subject consent and approvals from the Institutional Review Board of the University of Pittsburgh and the US Department of Defense Human Research Protection Office (ClinicalTrails.gov, identifier NCT01292876). Subjects were screened for established exclusion criteria.^14^ A total of 13 subjects were enroled and subjected to a custom designed physical therapy regimen both before and following implantation of one of three different xenogenic scaffold materials, all of which were composed of porcine ECM (Table 1). Patients were enroled in pre-operative physical therapy and required to reach a functional plateau before the surgical procedure so that post-operative improvements in function could not be attributed to therapy alone. Force production, functional task improvement, EMG analysis, CT or MRI imaging, and histology were used to evaluate return of strength, function and bioscaffold remodelling characteristics at 6–8 weeks, 10–12 weeks and 24–28 weeks post implantation.

### Subject selection and screening

Participants ranging from 18 to 70 years of age with a minimum 20% structural volume deficit as determined by MRI or CT, and/or 25% functional deficit of the muscle group mass when compared with the contralateral limb were eligible for inclusion in the study. All study subjects acquired VML at least 6 months prior to study inclusion. Exclusion criteria included poor nutrition, chronic disease, active infection, neoplasia, denervation or other medical comorbidities with the potential to impair wound healing.

Prior to inclusion in the trial, all subjects were screened by a licensed physical therapist to establish strength and functional deficits related to the anatomic location of interest, with respect to the contralateral limb. A detailed subject history was taken and the subject’s goals for participation in the study were recorded. Active and passive range-of-motion measurements were obtained at the joints both proximal and distal to the affected area using a goniometer. Isometric strength of the affected muscles was quantified using a hand-held dynamometer. Specific functional outcome variables were selected and evaluated for each subject based on their functional deficits and the objective measurements of strength and joint range-of-motion. Patient-reported outcomes, including the Disabilities of the Arm Shoulder and Hand (DASH)^29^ scale and Lower Extremity Functional Scale (LEFS)^30^ were administered, as appropriate. Subjects were also asked to provide a self-report of functional status at each of the tested time points. Outcome variables were established *a priori* for each subject through a study team consensus based on findings from the clinical examination specific to each subject and their observed strength and functional deficits. When possible, outcome variables were selected that were previously established as valid, reliable and aligned with the subject’s goals for the trial. Video recordings were performed during the evaluations when possible so as to ensure consistency in the testing positions across time points.

### Surgical procedure

All procedures were performed in a tertiary care medical centre under general anaesthesia, and tourniquet control of the extremity used. The injured muscle compartment was accessed, scar tissue was debrided and selective tenolysis performed. One of the following three ECM bioscaffolds was implanted at the site of missing muscle: MatriStem (ACell, Columbia, MD, USA); BioDesign (Cook Medical, Bloomington, IN, USA) or XenMatrix (C.R. Bard, Warwick, RI, USA) which were derived from porcine urinary bladder (UBM), SIS or dermis, respectively. All three scaffold materials were decellularized to meet established minimum criteria for DNA removal.^31^ MatriStem was used in the first six subjects, and the remaining seven subjects received either BioDesign or XenMatrix, randomly assigned. The ECM bioscaffold was cut to defect size-matched appropriate length and width, and implanted within the injury site with contact to adjacent native healthy tissue, and secured under tension with monofilament absorbable sutures. Care was taken to prevent folding or wrinkling and to ensure adequate soft tissue coverage. All empty space was closed before closure of the surgical site to ensure maximum scaffold-host tissue interaction, and a closed suction drain was placed.

### Physical therapy

#### Pre-surgical

Subjects were required to participate in rigorous pre-operative physical therapy for 4–16 weeks prior to surgery. The goal of the pre-operative physical therapy programme was to maximize performance with respect to the strength and functional outcome deficits identified during the screening examination. Due to the unique clinical presentation of each subject, physical therapy programs were customised for each subject to address the specific strength and functional deficits identified during the screening visit. Subjects were evaluated weekly on their progress by the treating physical therapist. Subjects were cleared to proceed to surgery after they reached a plateau in performance on their involved side, defined as functional gains of <2–3% over the course of any 2-week period, as determined by the treating physical therapist. The treating physical therapist was not a member of the investigative team. Outcome variables were tested by the same evaluating physical therapist who was a member of the investigative team at each time point.

#### Post-surgical

Post-surgical physical therapy was initiated between 24 and 48 h following surgery. No limitations were placed on the exercises or functional movements within the limits of tolerable pain. As early as the first post-operative day, targeted exercises were performed with the goal of stimulating muscle contraction and load bearing across the scaffold implantation site. Pain level, range-of-motion, strength and functional capacity were evaluated at each visit, and exercises were continued as tolerated. The post-operative physical therapy phase lasted 24 weeks.

### Isometric strength measurement

Isometric strength testing of the affected limb was measured 1–2 days prior to ECM implantation, and again at 6–8 weeks, 10–12 weeks and 24–28 weeks post-operatively. Measurements were taken using a hand-held dynamometer and standard manual muscle testing positions.^32^ Each measurement was repeated three times, and the average value of the three trials was calculated to represent as an indication of the isometric strength of the affected muscle.

### Range-of-motion and functional task analysis

Range-of-motion and functional task analysis was conducted pre-operatively and at 6–8 weeks, 10–12 weeks and 24–28 weeks post-operatively. All tasks were performed on both the affected and contralateral limb. Each task was repeated three times, and the average of the three trials was calculated as representative of performance on the task.

### Pre- and post-surgical imaging

Initial pre-operative CT imaging was performed on a 64-slice CT scanner (LightSpeed VCT, GE Healthcare, Chicago, IL, USA) at a slice thickness of 1.25 and 2.5 mm and a pitch of 1.375 in both bone and soft tissue algorithms. MRI protocols included a variety of sequences in sagittal, coronal and axial planes using T1-weighted spin echo, T2-weighted fast spin echo with or without fat suppression and STIR sequences. The kVp and mA were optimised with respect to the subject habitus and site imaged. Coronal and sagittal reformations were obtained. Three-dimensional volumetric reformatted imaging was also performed using Vitrea (Vital Images, Minnetonka, MN, USA) with surface rendering, as well as emphasis on the underlying musculature and osseous structures. Pre-operative imaging was reviewed by a musculoskeletal-trained radiologist (4 years’ experience). Initial CT imaging was assessed primarily for the presence of volumetric loss of bulk and/or fatty infiltration in the affected musculature. The overall percentage loss of muscle volume and severity of fatty infiltration was graded, where appropriate. Imaging was also evaluated for concomitant soft tissue (e.g., tendinous) and osseous injury. Post-operative imaging was performed at an ~7-month interval with similar imaging parameters. Post-operative imaging included characterisation of the location and appearance of the ECM scaffold, as well as a change in volume or appearance of the surrounding musculature. Overall percentage change in affected muscle volume was measured.

### Ultrasound-guided core biopsy of ECM

Ultrasound-guided biopsy of the surgically-placed ECM was performed ~6 weeks and 26 weeks post-operatively. Pre-procedural grayscale and colour/Power Doppler ultrasound of the operative site was performed to identify and characterise the surgically-placed ECM. After an appropriate needle trajectory was selected, the area was prepped and draped in sterile fashion. Local anaesthesia with skin infiltration and deeper injection was achieved with 1% lidocaine. Under ultrasound guidance, biopsy samples of the ECM bioscaffold and surrounding soft tissue were obtained using an 18-gauge spring-loaded biopsy needle (Temno, CareFusion, McGaw Park, IL, USA). A total of eight core samples were obtained at two separate biopsy sites. Biopsies spanned the proximal to distal length and medial to lateral width of the implantation site. Specimens were snap-frozen in liquid nitrogen and stored at −80 °C.

### Electrodiagnostic studies

As previously reported, nerve conduction and electromyography studies were performed for 8 of the 13 subjects using a Synergy EMG machine (Cardinal Health, Dublin, OH, USA).^33^ The specific nerve conduction studies completed and the specific muscles tested with needle examination were determined by location of the VML. Needle EMG analysis used concentric needle electrodes placed in the standard muscle belly and was performed at the proximal and distal site of the injured muscle if the standard muscle belly showed no evidence of electrical activity. Improvement in nerve conduction was defined as a ⩾20% increase in motor nerve conduction amplitude. For EMG studies, improvement was defined as either evidence of increased firing in volitional recruitment of muscles or a decrease in abnormal spontaneous activity compared with pre-operative results. Differences in amplitudes of CMAP were compared between pre- and post-ECM bioscaffold implant.

### Histology and immunolabelling

Frozen tissue sections were fixed in an ice cold 50:50 solution of methanol/acetone for 5 min and washed in phosphate-buffered saline (PBS). Tissue sections were incubated in blocking buffer to prevent non-specific antibody binding composed of 1% (w/v) bovine serum albumin (BSA), 2% (v/v) normal horse serum, 0·05% (v/v) Tween-20, 0·05% (v/v) Triton X-100 in PBS for 1 h at room temperature. Tissue sections were then incubated with primary antibodies diluted in blocking buffer as follows: mouse monoclonal CD146 (Abcam, Cambridge, MA, USA) at 1:350 and rabbit polycloncal Neurogenin-2 (NG2, Millipore, Billerica, MA, USA) at 1:200 as a perivascular stem cell markers, monocloncal anti-desmin (Abcam) at 1:200 for a muscle cell marker and (4) β-III tubulin at 1:200, for a neurogenic marker. After 16 h of incubation at 4 °C, tissue sections were washed with PBS and incubated with fluorophore-conjugated secondary antibodies (Alexa Fluor donkey anti-mouse 488 or 594 or donkey anti-rabbit 488, Invitrogen, Carlsbad, CA, USA) for 1 h at room temperature. After secondary incubation, nuclei were counterstained with 4′,6-diamidino-2-phenylindole (DAPI) and slides were coated with anti-fade mounting media (Dako, Carpinteria, CA, USA). Tissue sections were imaged using a Zeiss Axio-observer Z1 microscope using a ×20, 0.4 numerical aperture objective with a ×1.6 optovar magnification changer (Carl Zeiss, Oberkochen, Germany). Three fields of view were taken from each biopsy sample.

## Figures and Tables

**Table 1:** Patient information

*Subject*	*Age*	*Sex*	*Injury site (side)*	*Cause of injury*	*Months between injury and surgery*	*Number of previous surgeries*	*Tissue deficit (estimate)*	*Device used*
1	34	M	Anterior tibial compartment (left)	Exercise induced	13	5	58%	Acell, Matristem
2	37	M	Anterior tibial compartment (left)	Skiing accident	32	4	67%	Acell, Matristem
3	28	M	Quadriceps (left)	IED blast	18	14	68%	Acell, Matristem
4	27	M	Quadriceps (right)	IED blast	89	50	83%	Acell, Matristem
5	32	M	Anterior/lateral tibial compartment (left)	Skiing accident	85	8	90%	Acell, Matristem
6	31	M	Brachialis (left)	Wakeboarding accident	25	0	90%	Acell, Matristem
7	31	M	Biceps (right)	IED blast	86	8	33%	Cook, BioDesign
8	66	F	Quadriceps (left)	MVA	85	1	50.2%	Cook BioDesign
9	35	M	Quadriceps (right)	MVA	120	6	80%	Cook Biodesign
10	44	F	Rectus femoris (right)	Tendon rupture	7	2	48–56%	Bard, XenMatrix
11	31	M	Biceps/deltoid (left)	MVA	72	4	50%	Cook BioDesign
12	39	M	Sartorius (left)	Electrocution	12	11	25%	Cook BioDesign
13	30	M	Hamstring (left)	Sports injury	72	0	27%^a^	Bard, XenMatrix
Average	35.8				55.07	10.0	66.2%	
SEM	10.2				10.5	4.0	6.3	

Relevant information from each patient (*n*=13) included in the present study. Tissue deficit was estimated from MRI or CT scan. Data from patients 1 to 5 have been previously reported.^14^

Abbreviations: CT, computed tomography; IED, improvised explosive device; MRI, magnetic resonance imaging; MVA, motor vehicle accident

a Hamstring rupture resulted in proximal origin detatchment.

**Table 2:** Force production

*Subject*	*Injury site (side)*	*Activity*	*Baseline force measurement (lb)*	*6–8 weeks post surgery (%)*	*10–12 weeks post surgery (%)*	*24–28 weeks post surgery (%)*
1	Anterior tibial compartment (left)	Dorsiflexion		0.0	0.0	0.0
2	Anterior tibial compartment (left)	Dorsiflexion	0.0	0.0	0.0	0.0
3	Quadriceps (left)	Knee extension	6.0	*−10.0*	**18.3**	**20.0**
4	Quadriceps (right)	Knee extension	6.1	**127.9**	**149.2**	**136.1**
5	Anterior/lateral tibial compartment (left)	Dorsiflexion	3.6	*−33.3*	**16.7**	**33.3**
6	Brachialis (left)	Biceps flexion	35.8	NT	*−19.5*	*−17.9*
7	Biceps (right)	Wrist supination strength Biceps flexion	42.0 38.1	**66.7** **12.3**	**102.4** **7.6**	**126.2** **16.8**
8	Quadriceps (left)	Knee extension	10.3	**15.0**	**12.0**	**64.1**
9	Quadriceps (right)	Knee extension	33.3	**19.0**	**27.0**	**61.9**
10	Rectus femoris (right)	Knee extension	6.6	**11.0**	**30.0**	**86.4**
11	Biceps/deltoid (left)	Shoulder abduction Shoulder flexion Shoulder extension Elbow flexion Elbow extension	69.2 46.6 51.3 66.9 49.0	−4.6 **41.9** **13.3** 0.0 *−8.2*	*−4.1* **42.5** **22.6** *−0.3* **31.0**	**20.1** **104.1** **46.8** *−4.0* **1.6**
12	Sartorius (left)	Hip flexion Knee extension	68.1 92	NT	*−15.6* *−28.0*	*−3.5* *−1.1*
13	Hamstring (left)	Knee flexion Knee extension	53.5 99.2	**11.8** *−33.0*	**11.0** *−33.0*	*−3.4* **0.5**
			Average	**15.2**	**21.1**	**37.3**^**#**^
			s.e.m.	**12.6**	**12.2**	**12.4**

Strength measures as assessed with dynamometer from each patient presented as per cent change from pre-operation maximum after physical therapy. Bold and italicised text represents positive and negative changes, respectively. Data from subjects 1–5 obtained from previous report.^14^

Abbreviations: CT, computed tomography; MRI, magnetic resonance imaging; NT, not tested.

^#^indicates *P*<0.01 when compared with pre-operative values.

**Table 3:** Nerve conduction study of 8 out of 13 patients

*Subject*	*Evaluation site*	*Latency/amplitude (ms/mV)*		
		*Contralateral*	*Pre-op*	*Post-op*
1	Peroneal motor	2.5/3.7	2.6/3.7	2.5/2.5
2	Peroneal motor	2.7/6.8	2.8/2.5	2.5/**2.5**
3	Femoral motor	3.0/9.7	2.7/3.9	3.6/**4.8**
4	Femoral motor	NT/NT	3.1/10.9	3.6/4.8
5	Peroneal motor	3.7/10.0	2.3/1.7	2.1/1.5
7	Musculocutaneous motor	2.1/8.4	2.6/5.6	3.4/**6.9**
8	Femoral motor	2.5/7.2	1.2/3.8	2.9/**5.0**
9	Femoral motor	NT/NT	2.6/9.7	4.6/5.4

Four subjects showed an increase in compound motor action potential amplitude recorded in the targeted muscles: one in the tibialis anterior (Subject 2), two in the vastus medialis (Subjects 3 and 8) and one in the biceps brachii (Subject 7) indicated in bold. Subjects 3 and 8 showed an increase of CMAP amplitude of the femoral motor of >20% between pre-operative and post-operative time points. Subject 7 showed an increase in amplitude that is considered ‘normal’ when compared with the contralateral side.

Abbreviations: CMAP, compound motor action potential; NT, not tested.

**Table 4:** Needle electromyography shows improved recruitment patterns and disappearance of abnormal spontaneous activity

*Subject*	*Evaluation site*		*Pre-op*	*Post-op*
1	Tibialis anterior	ASA	−	−
		Recruitment	No unit	No unit
2	Tibialis anterior	ASA	−	−
		Recruitment	No unit	No unit
3	Vastus medialis	ASA	++++	**+++**
		Recruitment	No unit	No unit
	Vastus intermedius	ASA	++++	**+++**
		Recruitment	MD	GD
	Vastus lateralis	ASA	+++	+++
		Recruitment	GD	No unit
4	Vastus medialis	ASA	+++	+++
		Recruitment	No unit	No unit
	Vastus intermedius	ASA	++	**−**
		Recruitment	No unit	No unit
	Vastus lateralis	ASA	−	+
		Recruitment	Normal	Normal
5	Tibialis anterior	ASA	++	**−**
		Recruitment	GD	SD
	Extensor digitorum longus	ASA	++	**−**
		Recruitment	Single unit	SD
7	Biceps (proximal)	ASA	−	−
		Recruitment	Normal	Normal
	Biceps (distal)	ASA	NT	++
		Recruitment	NT	poly
8	Vastus medialis	ASA	+	**−**
		Recruitment	Normal	Normal
	Vastus intermedius	ASA	−	−
		Recruitment	Normal	Normal
	Vastus lateralis	ASA	−	−
		Recruitment	Normal	Normal
9	Vastus medialis	ASA	−	−
		Recruitment	Normal	Normal
	Vastus lateralis	ASA	−	−
		Recruitment	Normal	Normal
	Rectus femoris	ASA	−	−
		Recruitment	Normal	Normal

Four out of eight tested patients show disappearance of abnormal spontaneous activity in at least one tested muscle group (indicated in bold). Subject 5 showed a much improved recruitment pattern after surgery, compared with the baseline findings of generalised decreased recruitment pattern with a single motor unit firing in the EDL muscle. Overall, five of eight subjects improved electrophysiological function either in terms of increased CMAP (Table 3) or EMG profile. Data adapted from previous report.^33^

Abbreviations: ASA, abnormal spontaneous activity; CMAP, compound motor action potential; GD, greatly decreased; NT, not tested; SD, slightly decreased; −, not observed; ++, moderate numbers in three or more muscle areas.
