# Supplementary material for: An acellular biologic scaffold treatment for volumetric muscle loss: results of a 13-patient cohort study
Source: NPJ Regen Med. 2016 Jul 21;1:16008–. doi: 10.1038/npjregenmed.2016.8 (PMC5744714; doi:10.1038/npjregenmed.2016.8)
Supplement: Supplementary Information [file npjregenmed20168-s1.doc]

Supplemental Figure Legends.

**Table S1. Patient Eligibility Criteria**. Patients were screened against established exclusion and inclusion criteria

**Table S2. Functional task performance raw data for unreported patients.** Data presented as percent improvement from pre-surgical maximum (NT=not tested).

**Table S3. Range of motion.** Range of motion from each patient presented as a percent change from pre-operation maximum after physical therapy. Bold and italicized text represents positive and negative changes, respectively. Data from subjects 1-5 obtained from previous report.^14^ (NT = not tested), (# indicates p < 0.05 when compared to pre-operative values).

**Figure S1. Overview of study design**. Patients underwent 6-8 weeks of pre-operative physical therapy followed by up to 28 weeks of post-operative physical therapy. Tissue biopsies and functional assessments were evaluated 6-8, 10-12, and 24-28 weeks after ECM implantation.

**Figure S2. Representative gross changes of quadriceps following ECM implantation.** Gross appearance of injury site of patient 3 pre-operatively and 28 weeks post-ECM implantation

**Figure S3. ECM promotes muscle formation. (A,C,E)** Before surgery, at this level of the proximal aspect in the posterior compartment, patient 13 shows complete absence or atrophy of hamstrings. ECM implantation is associated with an increase in post-operative bulk muscle with areas measuring 5.4,6.9,and 7.3 cm^2^ at the proximal, distal, and middle aspect of the posterior compartment, respectively **(B,D,F).**
