# Supplementary figures and images for: An acellular biologic scaffold treatment for volumetric muscle loss: results of a 13-patient cohort study
Source: NPJ Regen Med. 2016 Jul 21;1:16008–. doi: 10.1038/npjregenmed.2016.8 (PMC5744714; doi:10.1038/npjregenmed.2016.8)

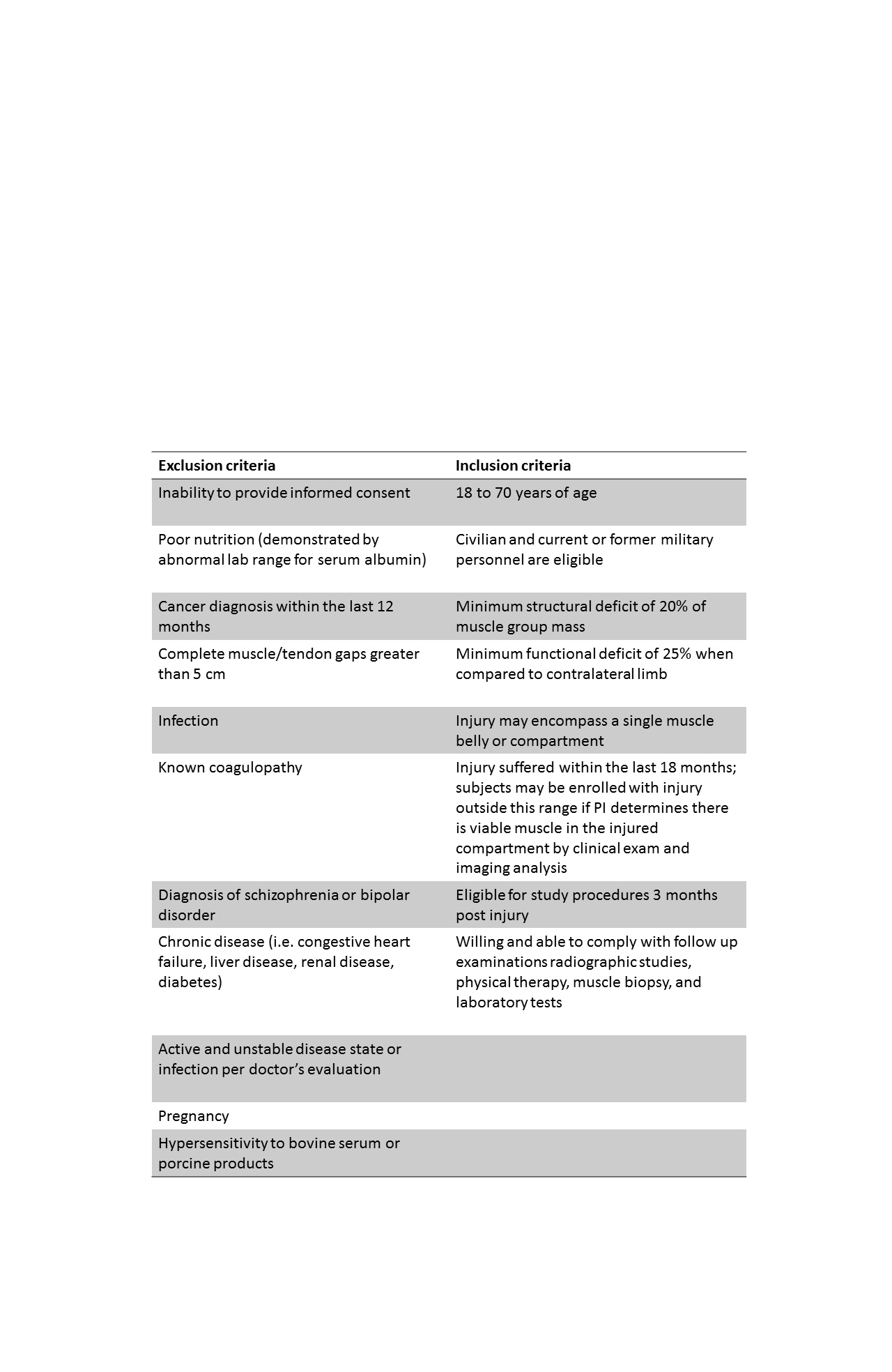

Supplement: Supplementary Figure S1 [file npjregenmed20168-s2.jpg]

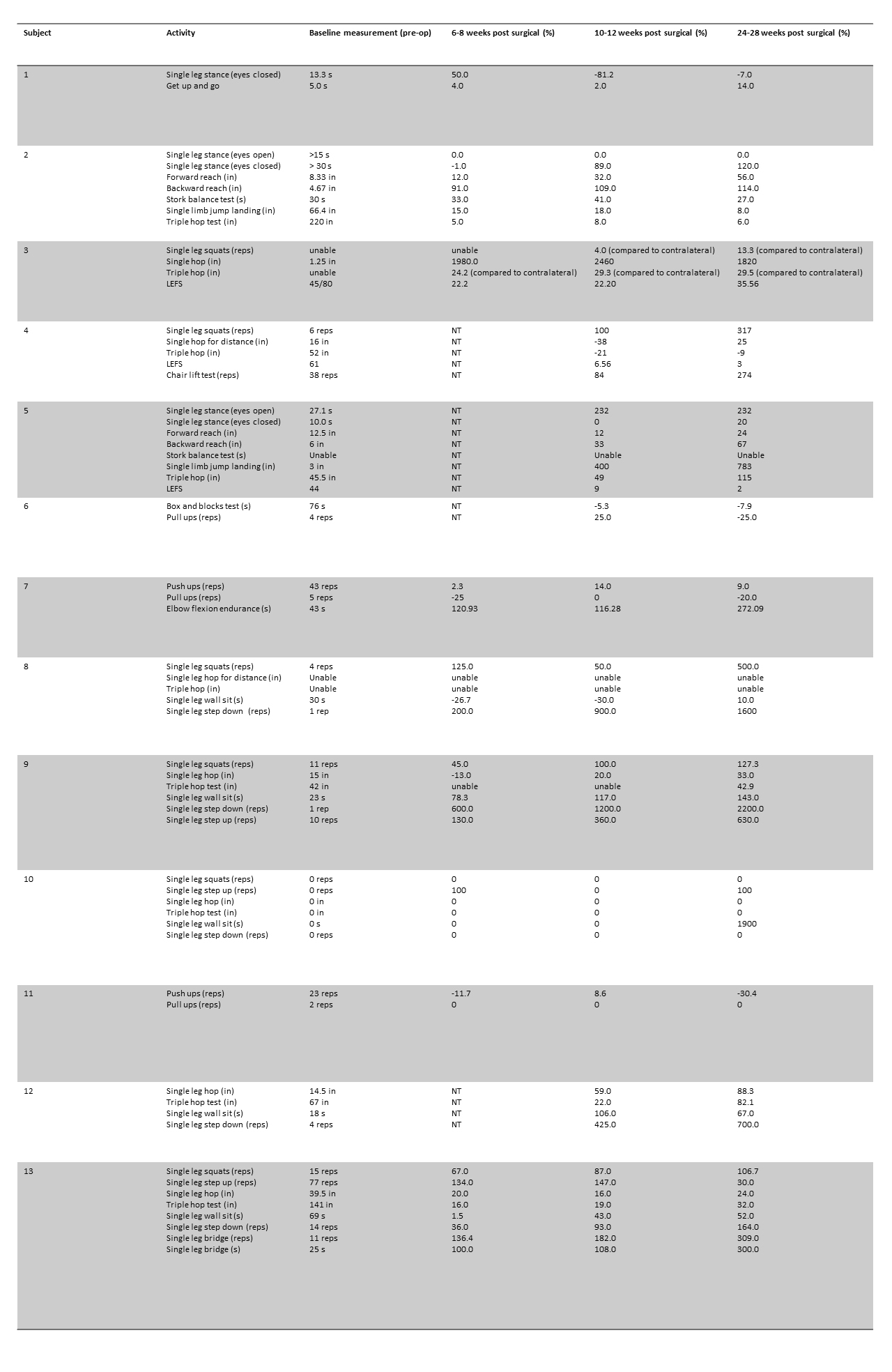

Supplement: Supplementary Figure S2 [file npjregenmed20168-s3.jpg]

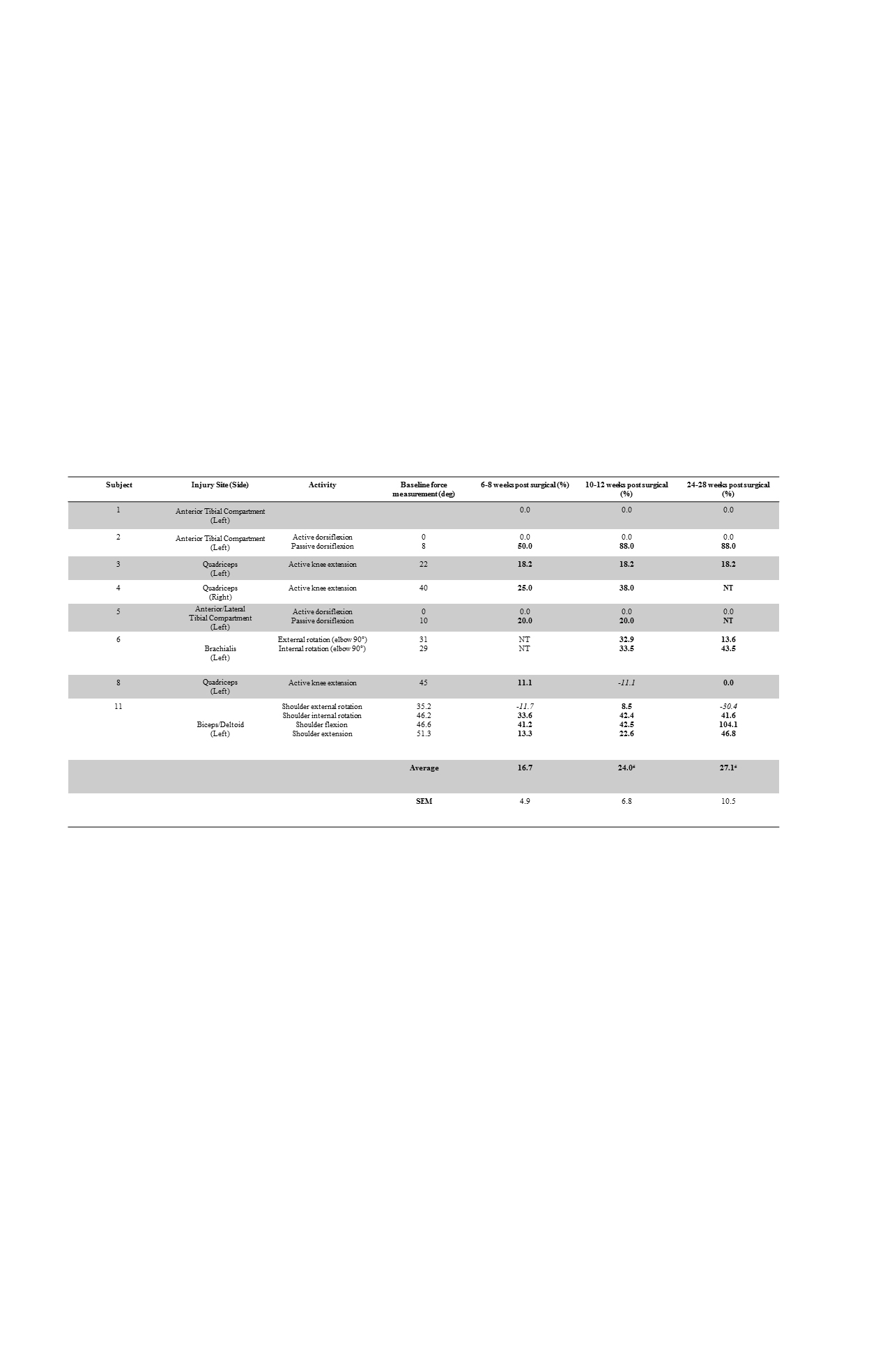

Supplement: Supplementary Figure S3 [file npjregenmed20168-s4.jpg]

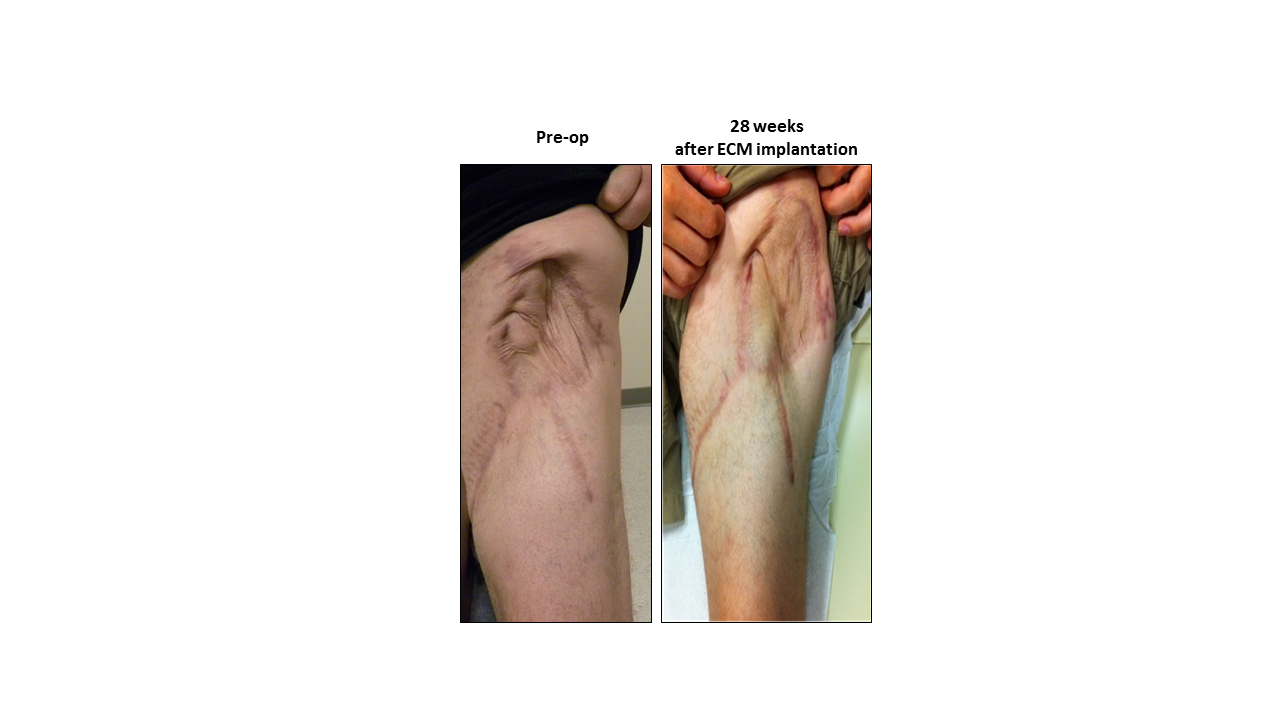

Supplement: Supplementary Table S1 [file npjregenmed20168-s5.jpg]

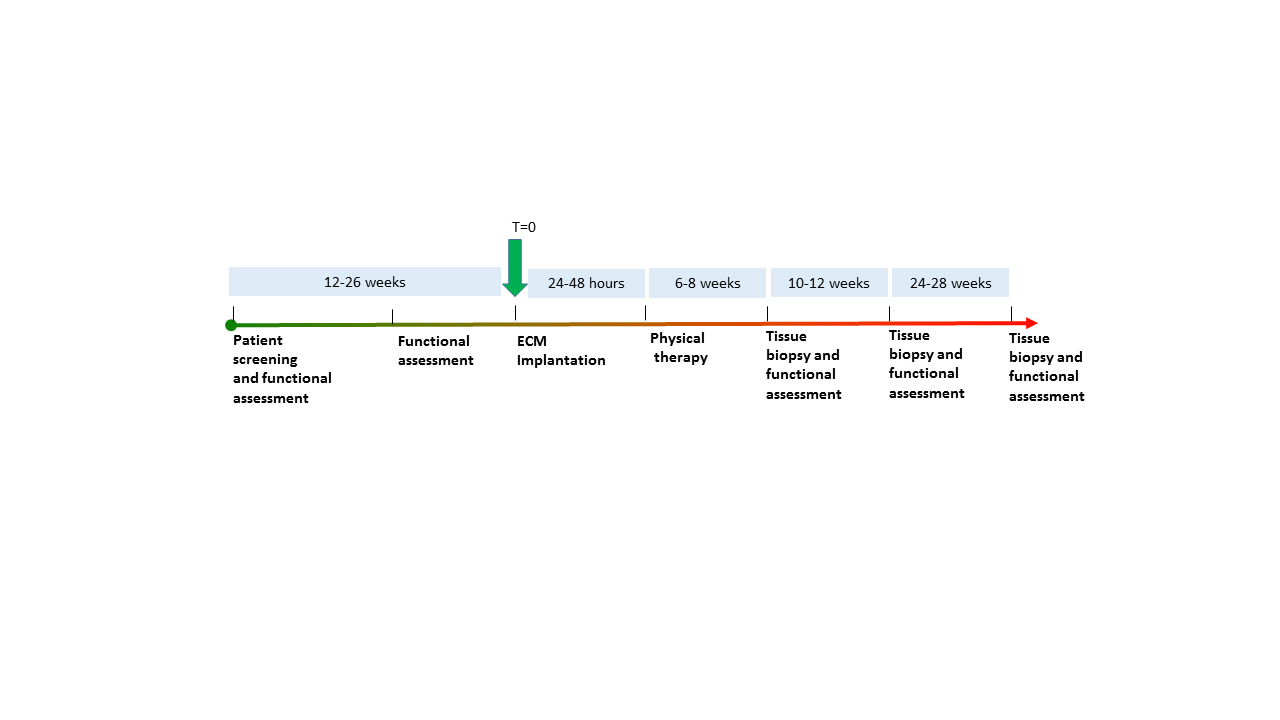

Supplement: Supplementary Table S2 [file npjregenmed20168-s6.jpg]

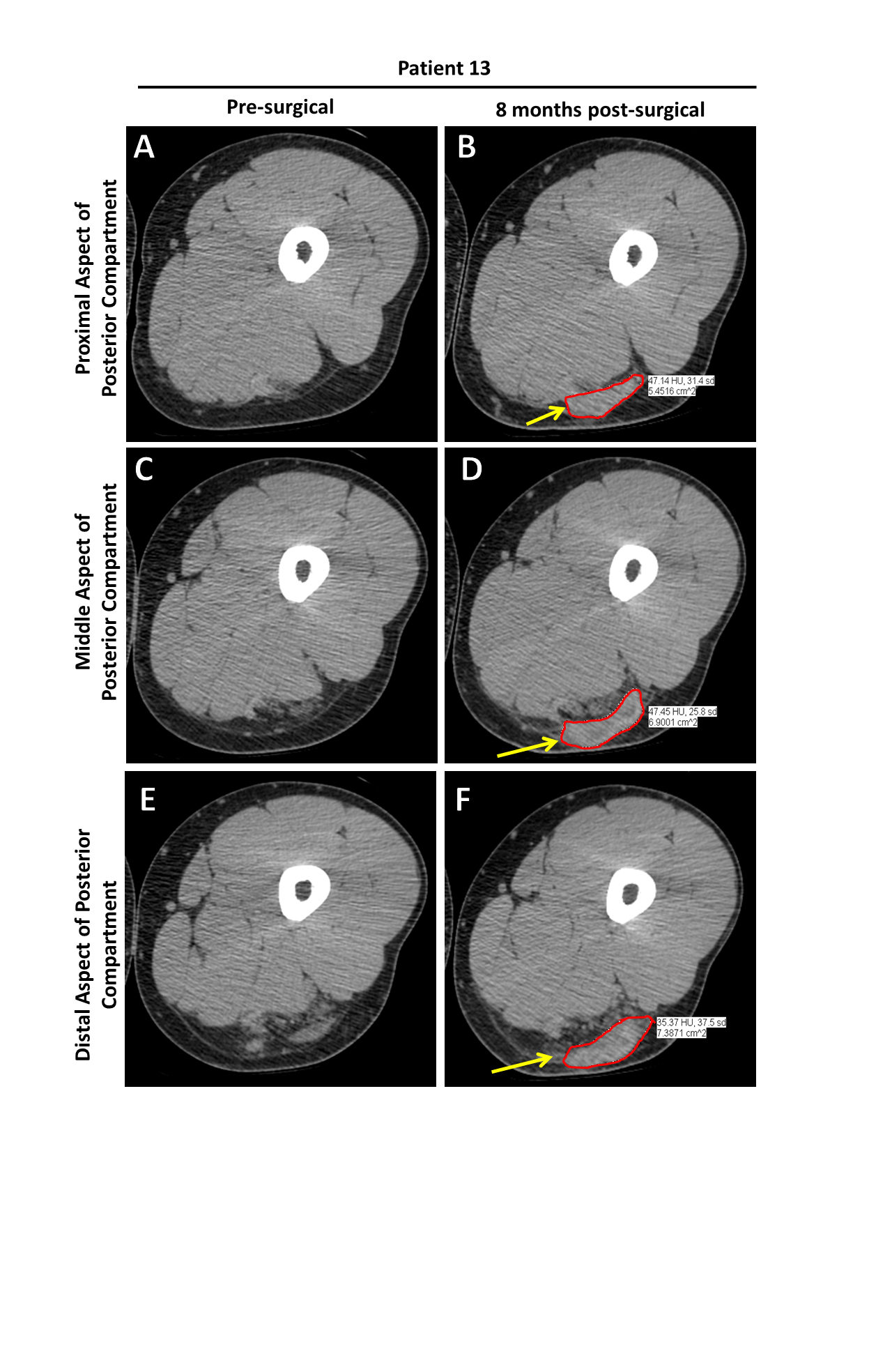

Supplement: Supplementary Table S3 [file npjregenmed20168-s7.jpg]
